# Unraveling the Enigma: A Report of a Rare Case of Acute Cord Syndrome Caused by Spinal Meningioma

**DOI:** 10.7759/cureus.48191

**Published:** 2023-11-02

**Authors:** Karam Rabi, Shams Alhammouri, Shadi Saa

**Affiliations:** 1 Department of Medicine, Faculty of Medicine & Health Sciences, An-Najah National University, Nablus, PSE; 2 Department of Neurosurgery, Palestinian Medical Complex, Ramallah, PSE

**Keywords:** dw-mri, neurological outcomes, emergency neurosurgery, acute cord syndrome, cns tumors, spinal meningioma

## Abstract

Spinal meningiomas (SMs) are a prevalent subtype of central nervous system tumors, with the majority adhering to the dura mater. In this case, we present the case of a 72-year-old female who initially reported numbness in her legs and the gradual onset of gait disturbances. Over a three-week period, these symptoms progressively worsened until she experienced a sudden onset of weakness and neurological deficits, leading to the diagnosis of acute cord syndrome (ACS). Magnetic resonance imaging revealed an anomaly within the extramedullary space, precisely located at the T8-T9 level. This anomaly exhibited peripheral gadolinium enhancement and demonstrated a dural tail sign, indicating the presence of an abnormal mass. Furthermore, a dorsal spine CT scan confirmed these findings by revealing a hyperdense lesion localized within the T8-T9 region. The lesion was situated posterior to the spinal cord, and conspicuous alterations in the coloration of the dura mater at the corresponding level were evident. A complete surgical resection was performed successfully, and the patient's surgical intervention proceeded without complications. Following the surgery, we observed significant improvements in both sensory and motor functions compared to the patient's preoperative state.

## Introduction

Acute cord syndrome (ACS) presents a neurological emergency marked by the abrupt onset of spinal cord dysfunction, often arising from spinal cord compression or ischemia [1]. One less common yet clinically significant cause of ACS is spinal meningiomas (SMs), which are prevalent neoplasms, accounting for 25-46% of all primary intracranial tumors [2]. They predominantly affect female patients, with an average age of approximately 50 years. While SMs can develop anywhere along the neuroaxis or spinal column, they are primarily observed in the thoracic (mid-spine) region (67-84%), the cervical spine (14-27%), and, rarely, in the lumbar spine (2-14%) [3]. SMs are typically found in the intradural and extramedullary regions, attaching to the dura mater. Despite being non-cancerous, the positioning of SMs within the spinal canal can lead to significant neurological impairments due to the compression of delicate neural tissue. Intradural spinal tumors are diagnosed at a rate of 64 cases per 100,000 person-years and constitute almost 3% of all primary tumors found within the central nervous system (CNS) [4,5]. Among these intradural spinal lesions, SMs are the second most common intradural lesions, surpassed only by spinal schwannomas [6].

The usual way SMs manifest clinically is with pain as the initial symptom, which is subsequently followed by issues related to walking, sensory function, and bowel or bladder control [7]. However, the clinical manifestation of SM often lacks specificity and can exhibit a range of symptoms, which may include either a chronic or abrupt compression of the spinal cord, leading to neurological dysfunction and the development of progressive myelopathy. Roughly 50% of individuals affected by this condition experience general back pain while radiating pain, motor deficits, and sensory loss tend to progress gradually over time [8].

This case report examines ACS caused by SM, offering insights into its clinical manifestations, diagnostic challenges, management complexities, and implications for patient outcomes. By shedding light on this uncommon yet potentially devastating condition, this case report aims to contribute to the growing body of knowledge surrounding ACS due to SM and underscores the importance of timely diagnosis and intervention in achieving favorable patient outcomes.

## Case presentation

**Figure 1:**
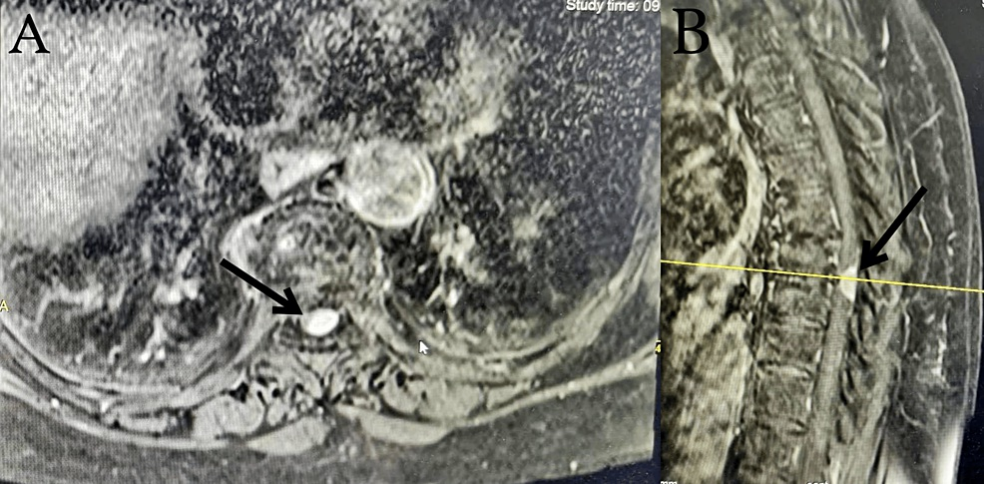
Preoperative MRI of the thoracic spine. A lobulated elliptical mass is seen in the T8-T9 dorsal space (black arrows). (A) Thoracic spine T1-weighted MRI transverse view. (B) Thoracic spine T1-weighted MRI sagittal view. MRI, magnetic resonance imaging.

A 72-year-old female was admitted complaining of persistent pain and numbness in her legs for three weeks and a recently developed gait disturbance. Additionally, the patient reported experiencing severe nocturnal back pain that appeared to have a crescendo pattern. However, the most striking observation was the acute onset of significantly exacerbated symptoms in the past 24 hours, rendering her entirely dependent on assistance for ambulation. There were no significant prior medical conditions or injuries in her history. A neurological assessment disclosed bilateral numbness in the lower limbs that was particularly left-side dominant. The patient exhibited a distinct sensory level on the right side, corresponding to the T10 dermatome. On the left side, the sensory level extended more cephalad, encompassing the dermatomal distribution up to T5. These findings suggest an asymmetrical sensory deficit, with a clear demarcation at the midline, and a higher level of sensory involvement on the left side compared to the right. Hip flexion and extension were motor grade 1/5 and 0/5 on the right side and left side, respectively. Knee flexion and extension were also motor grade 1/5 and 0/5 on the right side and left side, respectively. Ankle dorsiflexion and plantar flexion were motor grade 2/5 on the left side and 4/5 on the right side. Sensations were severely affected bilaterally, but more so on the left side. Bilateral Babinski and ankle clonus reflexes were positive. Bladder and anal sphincter functions remained intact, and there were no issues with the motor and sensory functions in the upper extremities. Plain radiography of the cervical and thoracic spine did not reveal any notable abnormalities. Magnetic resonance imaging (MRI) demonstrated a lobulated mass in the T8-T9 dorsal space, particularly in the intradural extramedullary space. T2-weighted MRI showed a low-signal-intensity elliptical lesion, which was enhanced peripherally on a gadolinium-enhanced T1-weighted image (Figure 1). The mass lesion caused compression and anterior displacement of the spinal cord.

**Figure 2:**
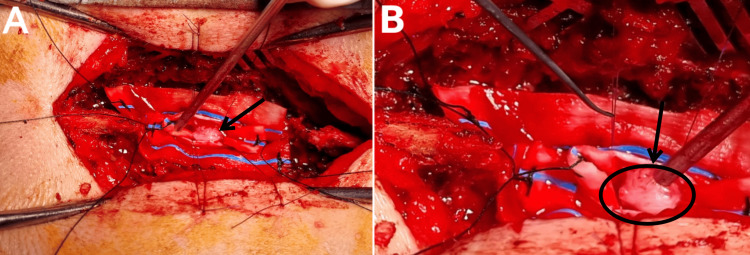
Intraoperative microscopic view. (A, B) Intraoperative microscopic view at different stages of operation. The tumor (black arrow) is noted to be covered with a grayish-white membrane and adherent to the dura (black circle).

The T7-T9 spinous processes were removed. A bilateral T8-T9 thoracic laminectomy and a vertical incision in the dura were performed. The arachnoid was dissected, and the lesion was visualized posterior to the cord. A microsurgical approach was employed to carry out meticulous resection and debulking (see Figure 2). A complete resection was successfully accomplished, and the specimen was subsequently forwarded for pathological examination.

**Figure 3:**
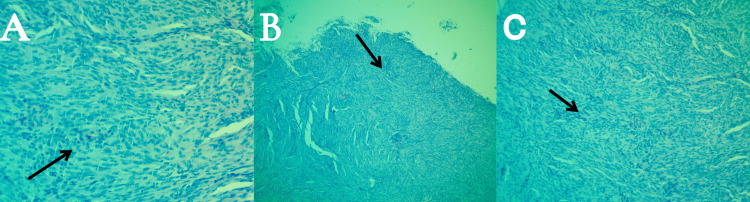
Microscopic views of the pathology specimen captured at varying levels of magnification. (A-C) Microscopic view of the pathology specimen showing the meningioma's distinctive histopathological features, including sheets of spindle-shaped tumor cells forming whorls (black arrows), indicative of a meningothelial meningioma subtype.

The histopathological examination of the tissue specimen, consisting of a single piece of grayish tissue measuring approximately 1 × 1 × 0.5 cm, definitively identified the presence of features in alignment with those indicative of a grade 1 meningioma, as depicted in Figure 3.

The leg numbness and gait disturbance displayed gradual improvement in the postoperative period. Upon assessing the patient postoperatively, we observed a remarkable enhancement in both lower limb motor and sensory functions compared to their preoperative state. Specifically, the motor strength in the right lower limb had progressed to grade 4/5, while the left lower limb demonstrated an equivalent motor grade of 4/5. This substantial improvement in motor function signifies a significant recovery in muscle strength in both lower extremities. Moreover, sensory evaluation revealed noteworthy improvements, indicating a substantial recovery of sensations that had been compromised before the surgery. This overall enhancement in motor and sensory functions postoperatively underscores the success of the intervention and the potential for an improved quality of life and mobility during the rehabilitation process.

One week following the surgical intervention, the patient's postoperative recovery has shown promising progress. Of particular note is the substantial improvement in the patient's gait disturbance, which has significantly ameliorated since the procedure. Encouragingly, the patient now exhibits the ability to move independently without requiring external assistance or support. This remarkable achievement underscores the favorable trajectory of the patient's postoperative rehabilitation, reflecting the effectiveness of the surgical intervention and providing optimism for continued recovery and improved mobility in the days ahead. A postoperative MRI was deemed unnecessary, as the surgical procedure successfully achieved complete resection of the lesion, with no indications or suspicions of any residual tumor remaining.

## Discussion

ACS is characterized by the sudden onset of neurological deficits resulting from spinal cord compression or ischemia [1]. In the context of an SM, the clinical presentation may vary but often includes symptoms such as severe back pain, muscle weakness, sensory disturbances, and autonomic dysfunction [9]. These symptoms may manifest differently depending on the tumor's location along the spinal cord. In our case, the patient presented with sensory and motor deficits, which prompted further investigation. Diagnosing ACS caused by an SM can be challenging due to its relative rarity and variable clinical presentation. Neuroimaging techniques, particularly MRI with contrast enhancement, play a crucial role in confirming the presence of an SM and assessing its impact on the spinal cord. In our case, MRI revealed the tumor's location and the extent of compression, which was instrumental in guiding treatment decisions.

The management of ACS caused by SM requires a multidisciplinary approach involving neurosurgeons, neurologists, and rehabilitation specialists. Prompt surgical intervention is the first-line therapy for SM and is generally considered a safe and efficient procedure in experienced centers. The rate of complete resection of SM mentioned in the literature ranges between 82% and 98% [10]. This intervention is often necessary to decompress the spinal cord and relieve pressure. The surgical approach may vary depending on the tumor's location, size, and relationship with adjacent structures. In some cases, radiation therapy may be considered an adjunctive treatment option, particularly when complete tumor removal is challenging [5]. Our case underscores the importance of timely surgical intervention, which led to significant postoperative improvements in both sensory and motor functions.

## Conclusions

The presented case demonstrates that ACS due to SM, though rare, demands swift recognition and intervention. When diagnosed early in its course and managed appropriately, patients have the potential to experience substantial improvements in neurological function, highlighting the critical role of multidisciplinary care and surgical expertise in optimizing patient outcomes. Further research and the sharing of clinical experiences will continue to refine our understanding and management of this complex condition, ultimately benefiting patients facing this challenging diagnosis.
